# Biocontrol Potential of *Trichoderma asperellum* Strain 576 against *Exserohilum turcicum* in *Zea mays*

**DOI:** 10.3390/jof9090936

**Published:** 2023-09-16

**Authors:** Yukun Ma, Yetong Li, Shijia Yang, Yu Li, Zhaoxiang Zhu

**Affiliations:** Engineering Research Center of Edible and Medicinal Fungi, Ministry of Education, Jilin Agricultural University, Changchun 130118, China; mahanhan0221@163.com (Y.M.); aliyetong@163.com (Y.L.); ysj18815819943@163.com (S.Y.); liyu@jlau.edu.cn (Y.L.)

**Keywords:** antagonistic ability, biocontrol agent, maize, cell wall degradation enzymes, seed germination

## Abstract

Maize is a crucial cereal crop in China, serving both as a staple food and an essential industrial resource. Northern corn leaf blight (NCLB) is a disease of corn caused by a fungus, *Exserohilum turcicum* (sexual stage *Setosphaeria turcica*). This study aimed to assess the biocontrol potential of various *Trichoderma* strains against *Exserohilum turcicum* 101 in Jilin, China. Through dual culture tests, the *Trichoderma* strains were categorized into four groups based on their antagonistic abilities. Eleven *Trichoderma* strains exhibited strong antagonistic behavior, with comparable or faster growth rates than *E. turcicum* 101. Microscopic observations confirmed that *T. asperellum* 576 hyphae effectively encircled *E. turcicum* 101 hyphae, reinforcing their antagonistic behavior. The production of non-volatile and volatile substances by the *Trichoderma* strains was evaluated, with *T. asperellum* 576 showing the highest potency in producing non-volatile and volatile substances, leading to an impressive 80.81% and 65.86% inhibition of *E. turcicum* 101 growth. Remarkably, co-culture suspensions of *T. asperellum* 576 + *E. turcicum* 101 and *T. atroviride* 393 + *E. turcicum* 101 exhibited strong antifungal activity. Furthermore, the activities of chitinase, β-1.3-glucanase, and cellulase were evaluated using the 3, 5-dinitrosalicylic acid (DNS) method. *T. asperellum* 576 + *E. turcicum* 101 displayed stronger cell wall degradation enzyme activity compared to *T. atroviride* 393 + *E. turcicum* 101, with values of 8.34 U/mL, 3.42 U/mL, and 7.75 U/mL, respectively. In greenhouse conditions, the application of a 10^7^ spores/mL conidia suspension of *T. asperellum* 576 significantly enhanced maize seed germination and plant growth while effectively suppressing *E. turcicum* 101 infection. Maize seedlings inoculated/treated with both *E. turcicum* 101 and *T. asperellum* 576 demonstrated substantial improvements compared to those inoculated solely with *E. turcicum* 101. The *T. asperellum* 576 treatment involved a 10^7^ spores/mL conidia suspension applied through a combination of foliar spray and soil drench. These findings highlight *T. asperellum* 576 as a promising biocontrol candidate against northern leaf blight in maize. Its antagonistic behavior, production of inhibitory compounds, and promotion of plant growth all contribute to its potential as an effective biocontrol agent for disease management.

## 1. Introduction

Maize (*Zea mays* L.) holds significant agricultural importance as a major crop in China [1]. It thrives in various regions, including the tropics, subtropics, and temperate areas, under both irrigated and rainfed conditions [2,3]. Maize stands out among other grains as a highly nutritious crop, providing essential nutrients for both humans and animals [4,5]. However, the devastating fungal disease Northern corn leaf blight (NCLB) is caused by the pathogen *Exserohilum turcicum* (Pass.) Leonard and Suggs (Pleosporaceae, Pleosporales, Pleosporomycetidae, Dothideomycetes, Pezizomycotina, Ascomycota, Fungi) [6,7,8] pose a significant threat to maize production. It occurs in all maize-producing regions, from tropical to temperate zones [9]. *E. turcicum* causes cigar-shaped green-grey lesions on leaves, which become necrotic in later infection stages and may evolve to blight symptoms, leading to high yield losses in maize [10]. Maize yield losses caused by *E. turcicum* are up to 50% in China from July to August every year. Uncontrolled NCLB can lead to substantial yield losses, documented at up to $2 billion in several countries [11,12]. With the projected increase in soil-borne pathogen species due to global warming, effective control of NCLB becomes even more critical, particularly for Asian countries like China. Traditionally, maize production practices heavily relied on chemical interventions, with three chemicals, metconazole, propiconazole, and prothioconazole, being the primary demethylation inhibitors and fungicides used in the production of corn fields [13]. However, the widespread use of these chemical substances poses risks to human health and the environment [14]. Therefore, it is essential to explore alternative or complementary control mechanisms and strategies to slow down the emergence of resistance in pathogens [15,16,17]. Biocontrol agents offer a promising avenue for crop protection. For instance, three *Bacillus* isolates demonstrated dominance and significantly reduced the growth rate of *E. turcicum* [18]. Among the biocontrol agents, *Trichoderma* strains present several advantages, including their ability to suppress multiple pathogens, their cost-effectiveness, and their promotion of soil fertility [19,20]. By utilizing biocontrol agents, the negative biological and environmental consequences associated with the continuous use of synthetic chemicals can be mitigated [21,22].

*Trichoderma* strains have gained significant attention as biological control agents (BCAs) since the groundbreaking research conducted by Weindling [23]. One of the key advantages of *Trichoderma* is its adaptability, as it can thrive in diverse soil types and tolerate a range of temperatures conducive to its growth [24,25]. *Trichoderma* exerts its protective effects on plants through various mechanisms, including mycoparasitism, antibiosis, promoting plant host resistance, production of plant growth-promoting substances, and modulation of plant hormonal pathways.

Mycoparasitism is a crucial attribute of *Trichoderma*, wherein it attacks other fungi by infiltrating their structures, killing them, and utilizing the nutrients within their cells [17]. This ability allows *Trichoderma* to compete effectively with other fungal pathogens for resources and space, thereby limiting their growth [26]. Additionally, *Trichoderma* exhibits a rapid growth rate, further aiding its competitiveness against plant infections. Antibiosis is another essential mechanism employed by *Trichoderma* in biological control. *Trichoderma* has the capacity to produce antimicrobial compounds that can inhibit the growth of pathogenic fungi [27]. These antimicrobial compounds include non-volatile compounds and volatile organic compounds (VOCs) [28]. Non-volatile compounds of *Trichoderma* have active effects against a range of plant pathogens [29]. Approximately 390 non-volatile compounds from several *Trichoderma* species have been summarized [30]. VOCs of *Trichoderma* were shown to inhibit plant pathogens, induce plant resistance, and directly promote plant growth, indicating that VOCs may play a role in the biocontrol activity of *Trichoderma* spp. [31,32,33]. As far, over 480 VOCs have been detected from *Trichoderma* species altogether. The detected *Trichoderma* VOCs comprise simple hydrocarbons, phenols, heterocycles, aldehydes, ketones, thioalcohols, thioesters, and their derivatives [34]. Research by Limdolthamand et al. demonstrated the efficacy of a fresh formulation of *Trichoderma harzianum* Rifai KUFA0710 in reducing the growth of the fungal pathogen *E. turcicum* in field trials [35]. This finding underscores the practical applicability of *Trichoderma* as a BCA in controlling plant diseases.

Furthermore, *Trichoderma* has been found to promote plant host resistance, offering an additional layer of protection against pathogens [36]. The exact mechanisms by which *Trichoderma* induces host resistance are not yet fully understood but have been demonstrated in various pathosystems [37]. The ability of *Trichoderma* to enhance plant defenses can contribute to long-term disease management and reduce the reliance on chemical pesticides.

The existing studies on *Trichoderma* as a BCA in the pathosystems of maize and *E. turcicum* are limited. Therefore, the primary objective of our investigation is to evaluate the effectiveness of forty-four strains of *Trichoderma* against *E. turcicum* through in vitro, in vivo, and seedling trials. This comprehensive approach aims to provide a more thorough understanding of *Trichoderma*’s potential as a BCA and its practical application.

## 2. Materials and Methods

### 2.1. Fungal Strains and Identification

Forty-four *Trichoderma* strains were collected from various provinces in China since 2014 (Table 1). The pathogenic strain *Exserohilum turcicum* was obtained from Jilin Agricultural University. All strains were preserved in the Engineering Research Center of the Chinese Ministry of Education for Edible and Medicinal Fungi at Jilin Agricultural University. The *Trichoderma* strains were isolated either by single ascospore isolation from fresh stromata of sexual morphs or by direct isolation from asexual morphs on the substrates [38,39]. The species identification of *Trichoderma* primarily relied on comprehensive morphological characteristics, including observations of stromata, ascus, and ascospores, as well as colony appearance, growth rate, and the structure of conidiophores and conidia. These morphological features were essential in distinguishing different *Trichoderma* species.

Genomic DNA was extracted from the mycelium of cultures on PDA using a Plant Genomic DNA Extraction Kit (TIANGEN Biosciences, Beijing, China). In cases where certain species presented challenges in identification, we employed molecular analysis of translation elongation factor 1-alpha (TEF1-α) and RNA polymerase II’s second largest subunit (RPB2) as an assisting method [40,41]. PCR products were cycle sequenced on an ABI 3730 XL DNA Sequencer (Applied Biosciences, Foster City, CA, USA) with primers reported by Jaklitsch [42] at Beijing Tianyihuiyuan Bioscience and Technology, China. The strains and the NCBI GenBank accession numbers of DNA sequences used in this work are listed in Table 1.

To support the species identification of *Trichoderma* strain 576, we conducted a comprehensive phylogenetic analysis. Sequences were assembled, aligned, and manually adjusted when needed with BioEdit 7.0.5.3 [43]. NEXUS files were generated with Clustal X 1.83 [44]. To achieve accurate placement within the phylogenetic framework, we utilized both TEF1-α and RPB2 sequences in our analyses. We selected a set of 18 *Trichoderma* taxa, including *T. thelephoricola* 342, as an outgroup taxa.

Maximum likelihood (ML) analysis was performed with RaxmlGUI 2.0 [45], the ML + rapid bootstrap setting, and the GTRGAMMAI substitution model with 1000 bootstrap replicates. Analyses were performed with all characters treated as unordered and unweighted and gaps treated as missing data. Topological confidence of resulted trees was tested by maximum parsimony bootstrap proportions (MLBP) with 1000 replications, each with 10 replicates of random addition of taxa. MLBP greater than 50% is shown at the nodes.

### 2.2. Dual-Culture Antagonistic Activity Assay

To assess the antagonistic effect of the *Trichoderma* strains against *E. turcicum* 101, a dual culture test was conducted. Based on the observations of *Trichoderma*’s growth rate, we decided to introduce the *E. turcicum* 101 plugs onto the dual culture agar two days prior to the addition of *Trichoderma*. This time interval allowed the *E. turcicum* 101 isolates to establish themselves before encountering *Trichoderma*. The procedure involved placing 5-mm diameter fungal plugs of both the *Trichoderma* strains and *E. turcicum* 101 on Petri dishes (90 mm diameter) containing potato dextrose agar (PDA), with a distance of 5 cm between them [46]. The Petri dishes were then incubated at 25 °C under a 12-h cycle of light and dark for a period of 15 days. The experimental design followed a completely randomized design, with three replications performed. Each experimental iteration was repeated three times to ensure reliable and consistent results. The growth rate and sporulation of *Trichoderma* species on *E. turcicum* 101 were monitored and documented on the fourth, seventh, and fifteenth days after inoculation using a Canon G5 digital camera (Canon, Tokyo, Japan). The findings were analyzed based on the degree of antagonism observed. Zhang et al. utilized four categories to describe the degree of antagonism: strong antagonism, moderate antagonism, weak antagonism, and absence of antagonism [47]. In the case of *Trichoderma* strains exhibiting strong antagonism, they displayed rapid growth comparable to or even faster than phytopathogenic fungi. Additionally, they formed extensive hyphal coilings around the hyphae of the pathogenic fungi and exhibited abundant sporulation on the colonies of the phytopathogenic fungi within a span of 15 days. *Trichoderma* strains demonstrating moderate antagonism also exhibited fast growth rates similar to phytopathogenic fungi. They were capable of sporulating on the colonies of phytopathogenic fungi within 15 days, although the sporulation may be comparatively less abundant. On the other hand, *Trichoderma* strains with weak antagonism tended to grow slower than phytopathogenic fungi. These strains displayed minimal or no sporulation on the phytopathogenic fungal colonies within the 15-day timeframe. Finally, *Trichoderma* strains lacking antagonism exhibited limited and slow growth, often becoming overgrown by the colonies of phytopathogenic fungi. These categories were used to assess and interpret the results obtained from the dual culture test.

### 2.3. Effect of Non-Volatile Substances Produced by Trichoderma Strains

The *Trichoderma* strains and *E. turcicum* 101 were initially inoculated on PDA and incubated at a temperature of 25 °C. The cellophane filtration membrane method, originally developed by Dennis and Webster, was employed to assess the ability of a subset of *Trichoderma* strains to generate non-volatile inhibitors [29]. The procedure involved inoculating the *Trichoderma* strains onto cellophane-covered Petri dishes, which were then incubated for 4 days at 25 °C. After removing the cellophane and mycelium, an *E. turcicum* 101 inoculum Petri dish was placed in the center of the medium previously occupied by *Trichoderma* strains. Following a 7-day reincubation at 28 °C, the inhibitory rate of the *Trichoderma* strains was determined by calculating the difference in diameter between the target colony in the absence and presence of the antagonist. This experiment was repeated three times. The inhibitory rate of the *Trichoderma* strains was calculated using the provided formula:

Inhibition rate (%) = (D1 − D2)/D1 × 100(1)

where D1 is the diameter of the target colony in the absence of the antagonist, while D2 is the diameter of the target colony in the presence of the antagonist.

### 2.4. Effect of Volatile Substances Produced by Trichoderma Strains

*E. turcicum* 101 were initially inoculated on PDA and incubated at a temperature of 25 °C for 3 days. The *Trichoderma* strains were initially inoculated on PDA and incubated at a temperature of 25 °C for 1 day. After one day, the covers of the Petri dishes were replaced with the bottoms of 3-day-old PDA cultures of *E. turcicum* 101, as described by Jin and Khalid [48]. The two halves or cultures of the plates were securely taped together using parafilm tape and maintained at 25 °C for a duration of 10 days. In the control group, only *E. turcicum* 101 was inoculated. To ensure standardized *Trichoderma* sporulation, all bioassay procedures were conducted under controlled light-limited conditions [49]. Each test was repeated three times to ensure accuracy, and the inhibition of mycelial radial growth was calculated as described previously.

### 2.5. Fermentation Broth Antagonistic Assays

In order to assess the interaction between *Trichoderma* strains and *E. turcicum* 101 and evaluate the mycelial radial growth inhibition, the following experimental procedure was conducted. *E. turcicum* 101 was cultured on PDA plates at a temperature of 30 °C for 7 days. Meanwhile, *Trichoderma* strains were grown on PDA plates at 25 °C for 5 days. Circular five mycelia blocks with a diameter of 5 mm were created at the edge of the colonies using a punch. The five mycelia blocks of both *Trichoderma* strains and *E. turcicum* 101 were inoculated into sterilized potato dextrose broth (PDB) medium in the same flasks together. The flasks were then placed on a shaker incubator and incubated for 10 days at a temperature of 28 °C with a shaking speed of 150 rpm. From day 2 to day 12 of the fermentation process, 10 mL of the liquid culture was extracted daily. The fermented liquid was filtered using filter paper and subsequently centrifuged at 12,000 rpm for 10 min. Finally, it was filtered with a 0.22 μm filter membrane [50]. The resulting fermented liquid was mixed with solid PDA medium (approximately 60 °C) in a proportion of 1:9 (*v*/*v*) and poured into plates. A 5 mm diameter block of *E. turcicum* 101 was placed in the center of each plate, and the plates were incubated at 25 °C for 5 days. Each treatment was replicated three times. The mycelial radial growth inhibition was calculated as described previously.

### 2.6. Enzymatic Activity

To evaluate the activities of chitinase, β-1.3-glucanase, and cellulase, a co-culture suspension was prepared as described in 2.5. Colloid chitin, β-1,3-glucan, and Sigma-Aldrich (CMC) were utilized as substrates to measure the respective enzyme activities. The assays for all three enzymes were conducted using the 3,5-dinitrosalicylic acid (DNS) method developed by Miller [51]. For the chitinase assay, the reaction mixture comprised a co-culture supernatant, which was added to 200 mL of 0.5% colloidal chitin in 50 mM sodium acetate buffer (pH 5.0). The mixture was incubated at 37 °C for 1 h. To stop the reaction, 500 µL of DNS reagent was added, followed by boiling for 5 min. In the β-1.3-glucanase assay, the reaction mixture consisted of 100 μL of 0.2 mM sodium acetate buffer (pH 5.5), 50 μL of enzyme extract, and 50 μL of laminarin substrate. The mixture was incubated at 37 °C for 30 min. The reaction was stopped by adding 500 µL of DNS reagent and boiling for 5 min. For the cellulase assay, the reaction mixture included 100 mL of 50 mM sodium acetate buffer (pH 5.0), 50 mL of 1% CMC substrate, and 50 mL of enzyme extract. The mixture was incubated at 50 °C for 60 min. The reaction was terminated by adding 500 µL of DNS reagent, followed by boiling in a water bath for 5 min. The spectrophotometer recorded absorption peaks at 585 nm, 540 nm, and 620 nm to measure the enzymatic activities of chitinase, β-1.3-glucanase, and cellulase, respectively. The experiment was replicated three times for each treatment, and the entire study was repeated at least three times to ensure reliability.

### 2.7. Effect of T. asperellum 576 on Maize Seed Germination

To assess the germination effects of *T. asperellum* 576, the maize seeds were subjected to surface disinfection using a 1.5% sodium hypochlorite solution for 5 min [52], followed by placement in clear plastic boxes with lids. Initially, *T. asperellum* 576 cultures were grown on a PDA medium. After the formation of spores, sterile distilled water was added to the PDA plate. The mycelium was gently rubbed with a sterilized needle to release the spores into a centrifugal tube, which was then collected from the plate using a micropipette to obtain mycelium spores. Using a hematocytometer, the number of conidia was determined, and their concentration was adjusted to 10^7^ spores/mL. The conidial suspension was placed in a centrifugal tube. Each treatment group consisted of 30 seeds: one group was treated with 10 mL of 10^7^ spores/mL *T. asperellum* 576 conidial suspension, while the control group was treated with 10 mL of sterile distilled water. The seeds were placed on moistened blotter papers inside the plastic boxes after surface disinfection. Subsequently, 10 mL of either *T. asperellum* 576 conidial suspension or sterile distilled water was poured into each box. The boxes were then incubated at 25 °C with a 16-h light cycle, and the germination progress was monitored every 12 h. The experiment was replicated three times for each treatment, and the entire study was repeated at least three times to ensure reliability. Germination was recorded when the radicle protruded through the seed coat.

### 2.8. Biocontrol Effect of T. asperellum 576 against E. turcicum 101

Maize seeds used in this study were sourced from the Jilin Provincial Agricultural Science laboratory. The seeds were sown in pots filled with sterile soil consisting of a 1:1 ratio of vermiculite to soil [37]. The pots had dimensions of 14 cm in diameter and 12 cm in height. The seedlings were grown in a greenhouse with controlled conditions, maintaining a temperature of 25 °C and relative humidity of 85%. At the beginning of the experiment, all seedlings were at the four-leaf stage.

To evaluate the biocontrol effect of *T. asperellum* 576 against *E. turcicum* 101 under greenhouse conditions, maize seedlings were subjected to four treatments. These included: (T1) plants without *T. asperellum* 576 and *E. turcicum* 101, (T2) *T. asperellum* 576-treated plants, (T3) *E. turcicum* 101-inoculated plants, and (T4) *E. turcicum* 101 and *T. asperellum* 576-inoculated/treated plants. The seedlings were cultivated under controlled conditions with a temperature of 25 °C, a 12-h light/dark cycle, and 60–70% humidity. They were watered every two days for a period of 30 days. 

T1: a control group treated with 30 mL of sterile distilled water; T2: seedlings treated with a combination of foliar spray (30 mL) and soil drench (30 mL) application of conidial suspension of *T. asperellum* 576, containing 10^7^ conidia/mL; T3: seedlings treated with a foliar application of 30 mL conidial suspension of *E. turcicum* 101, containing 10^5^ conidia/mL, to induce infection; and T4: seedlings treated with a foliar application of 30 mL conidial suspension of *E. turcicum* 101 and subsequently treated with *T. asperellum* 576 using a combination of foliar spray (30 mL) and soil drench (30 mL) methods, both with conidial suspension containing 10^7^ conidia/mL. The experiment followed a completely randomized design with a total of sixty seedlings (four treatments × fifteen replicates), and the entire experiment was conducted three times.

After a treatment period of 20 days, the seedlings were visually examined for symptoms or signs of infection by *E. turcicum* 101 and compared with the control group. Plant height was measured from the base of the seedling to the tip of the tallest shoot using a vernier caliper with an accuracy of 0.1 mm. Stem diameter was measured at the midpoint of the main stem using the same vernier caliper. Fresh weight measurements of stems and roots were recorded using an electronic balance with a precision of 0.01 g immediately after harvesting. Subsequently, the plant samples were dried in an oven at 100 °C for 30 min to remove surface moisture and then dried at 80 °C for 48 h, or until a consistent mass was achieved, to determine the dry weight.

### 2.9. Statistical Analysis

Statistical analyses were conducted using Minitab^®^ Statistical Software version 18 (IBM Co., New York, NY, USA). The data were analyzed using analysis of variance (ANOVA), followed by Fisher’s least significant difference (LSD) multiple comparison test (*p* < 0.05) to determine significant differences between treatment groups. The results are presented as means of replicates or independent experiments, and the error bars represent the standard deviation (SD).

## 3. Results

### 3.1. Species Identification and Phylogenetic Analysis

**Figure 1:**
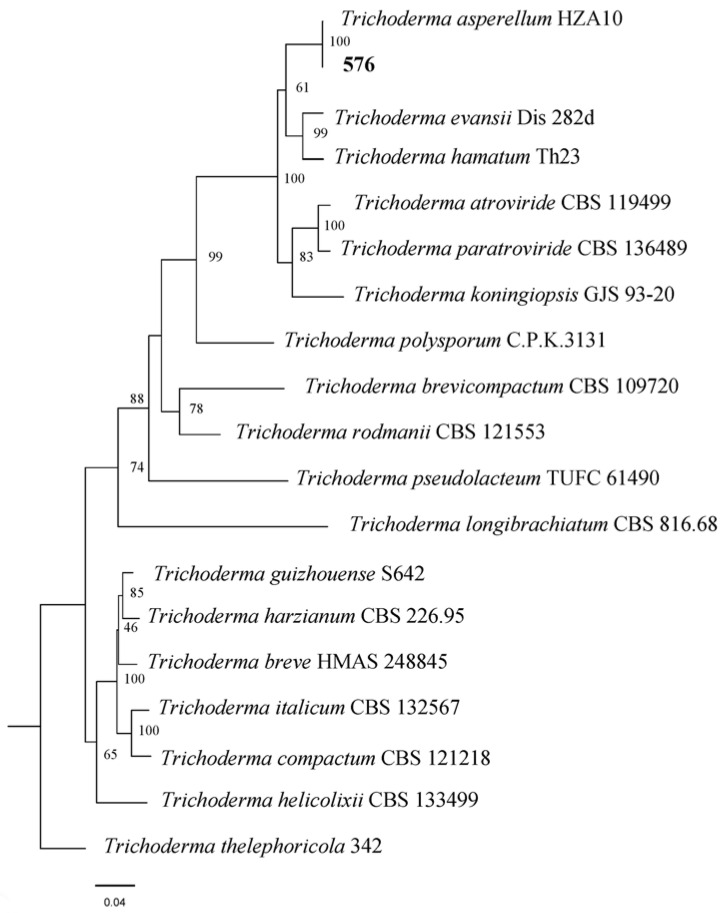
Maximum likelihood tree showing the evolutionary relationships among different *Trichoderma* species based on translation elongation factor 1 alpha (TEF1-α) gene and the RNA polymerase II second largest subunit (RPB2) sequences. Maximum likelihood bootstrap values above 50% are indicated at nodes. The tree is rooted with *Trichoderma thelephoricola* 342. The species in this study are indicated in boldface.

Employing a comprehensive analysis method that integrates both morphology and molecular sequences, we successfully identified a total of 44 distinct *Trichoderma* strains (Table 1). All accompanying plate photographs are available in Supplementary Materials Figure S1. The subsequent phylogenetic analysis involved 18 selected taxa representing major *Trichoderma* species. The alignment of sequences facilitated the definitive classification of isolate 576 as *Trichoderma asperellum*, as evident in the phylogenetic tree (Figure 1).

### 3.2. Dual Culture Tests

**Figure 2:**
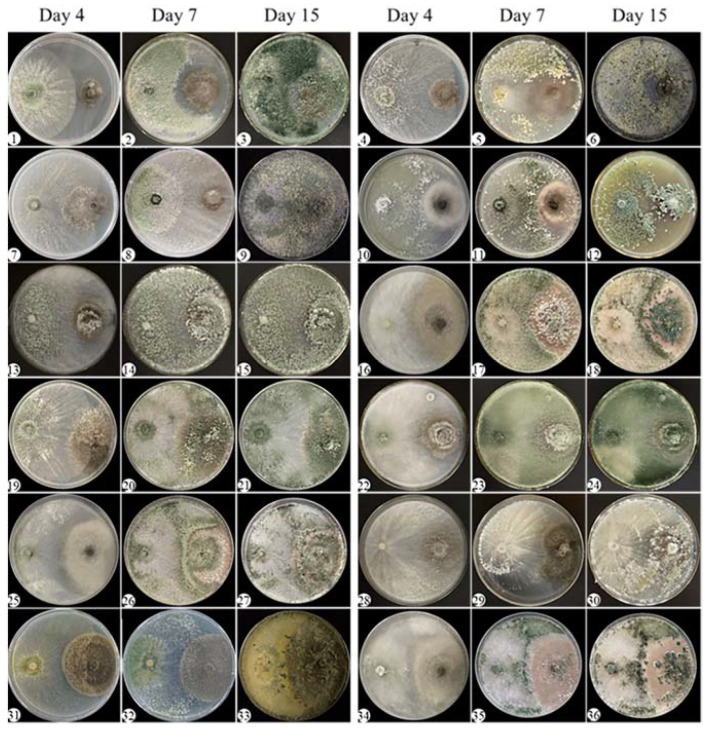
Dual culture assays of *Trichoderma* strains with strong antagonistic and *Exserohilum turcicum* 101. The left side shows *Trichoderma* strains and the right side shows *E. turcicum* 101. 1–3: 576; 4–6: 393; 7–9: 64; 10–12: 3A; 13–15: 342; 16–18: 285; 19–21: TU2; 22–24: XZ1-3; 25–27: 402; 28–30: MSL-3; 31–33: 204; 34–36: 417. The images for the fourth, seventh, and fifteenth days of co-culture are arranged from left to right.

**Figure 3:**
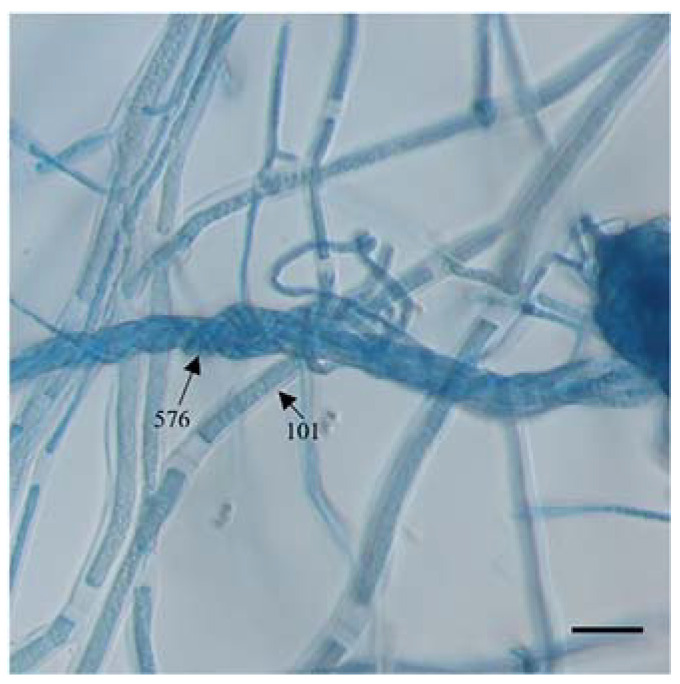
Observation of the interaction zone between *Trichoderma asperellum* 576 and *Exserohilum turcicum* 101. Scale bars = 20 μm.

Among the selected strains, *Trichoderma* strains 576, 393, 64, 3A, 342, 285, TU2, XZ1-3, 402, MSL-3, and 204 displayed strong antagonistic behavior. These strains exhibited comparable or faster growth rates than *E. turcicum* 101, formed extensive hyphal coilings around the hyphae of *E. turcicum* 101, and showed abundant sporulation on the colonies of *E. turcicum* 101 over a 15-day period (Figure 2). Microscopic observations provided visual evidence of the interaction between *T. asperellum* 576 and *E. turcicum* 101, showing the encirclement of *E. turcicum* 101 hyphae by *T. asperellum* 576 hyphae (Figure 3). The mycelium of *Trichoderma* was observed to be entangled in the mycelium of *E. turcicum* 101. This interaction may imply that *Trichoderma* parasitizes the mycelium of *E. turcicum* 101, or it may be part of a competitive process by which *Trichoderma* inhibits the growth of the pathogen. This observation further supports the antagonistic behavior and potential biocontrol activity of the identified *Trichoderma* strains.

In addition, twelve *Trichoderma* strains, namely 110, 224, 295, 375, 99, 421, 539, 581, 593, 526, 578, and XZ9-1, demonstrated a moderate antagonistic response after a 15-day co-culture period. These strains exhibited accelerated growth rates comparable to or surpassing that of *E. turcicum* 101 but displayed limited sporulation on the colonies of *E. turcicum* 101 (Supplementary Materials Figure S2). The strong and moderately antagonistic *Trichoderma* strains hold promise for biocontrol applications and warrant further comprehensive investigation and research.

On the other hand, weakly antagonistic *Trichoderma* strains had a decelerating effect on the growth of *E. turcicum* 101. These strains caused limited sporulation of *E. turcicum* 101 colonies within the 15-day co-culture period (Supplementary Materials Figure S3).

The results also revealed that *Trichoderma* strains did not exhibit detectable antagonistic behavior towards *E. turcicum* 101. These strains displayed slower and restricted growth rates compared to *E. turcicum* 101, suggesting a limited potential for biological control measures (Supplementary Materials Figure S4).

### 3.3. Antagonistic Effect by Trichoderma Non-Volatile Substances

**Figure 4:**
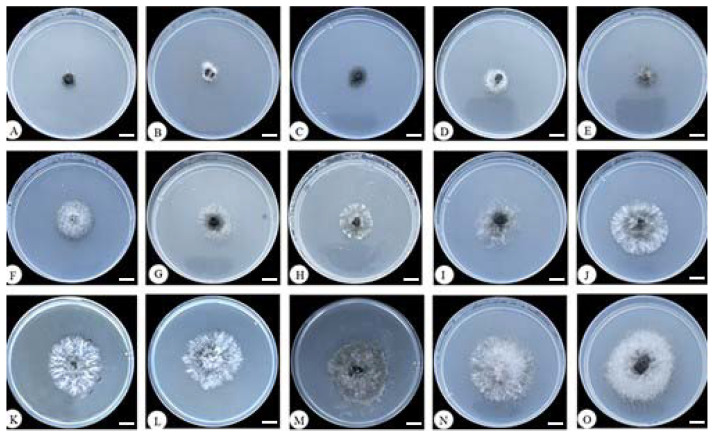
Inhibition effect of non-volatile substances of *Trichoderma* strains against *Exserohilum turcicum* 101. (**A**) 576; (**B**) 393; (**C**) 421; (**D**) 110; (**E**) 3A; (**F**) XZ9-1; (**G**) 285; (**H**) 539; (**I**) 417; (**J**) XZ1-3; (**K**) 342; (**L**) TU2; (**M**) 64; (**N**) 402; (**O**) CK. Bars = 10 mm.

According to Table 2 and Figure 4, *T. asperellum* 576 exhibited the highest potency in producing non-volatile compounds, resulting in an inhibition of *E. turcicum* 101 growth by 80.81%. Additionally, seven strains (393, 421, 110, 3A, XZ9-1, 285, and 539) showed inhibition rates over 50.00%, while six strains (417, XZ1-3, 342, TU2, 64 and 402) exhibited less than 50.00% inhibition against *E. turcicum* 101. These results provide valuable insights into the antifungal potential of the examined *Trichoderma* strains and highlight *T. asperellum* 576 as a particularly promising candidate for further investigation and utilization in biocontrol strategies.

### 3.4. Fungal Growth Inhibition by Trichoderma Volatile Substances

**Figure 5:**
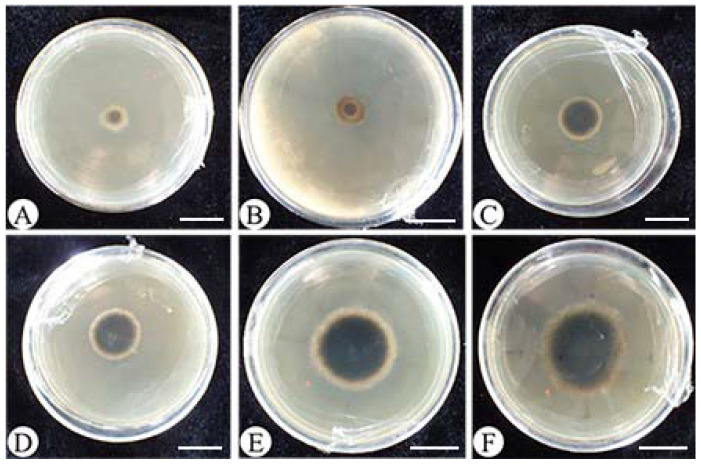
Inhibition effect of volatile substances of *Trichoderma* strains against *Exserohilum turcicum* 101. (**A**) 576; (**B**) 393; (**C**) 421; (**D**) 110; (**E**) 3A; (**F**) CK. Bars = 20 mm.

As shown in Table 3 and Figure 5, *T. asperellum* 576 displayed the highest inhibitory effect on *E. turcicum* 101 mycelial growth with a rate of 65.86% compared to CK. Following *T. asperellum* 576, *Trichoderma* strains 393, 421, 110, and 3A also showed considerable inhibitory activity, with inhibition rates ranging from 23.10% to 57.54%. Based on these findings, *T. asperellum* 576 and *T. atroviride* 393 were selected for further testing and investigation, given their strong potential as biocontrol agents against *E. turcicum* 101.

### 3.5. Antifungal Activity of the Co-Culture Suspension against E. turcicum 101

According to Table 4, the co-culture suspensions of *T. asperellum* 576 + *E. turcicum* 101 or *T. atroviride* 393 + *E. turcicum* 101 exhibited strong antifungal activity against *E. turcicum* 101, as evidenced by the reduction in mycelial growth over time. All co-culture suspensions demonstrated mycelial inhibition ranging from 12.48% to 64.45%. Specifically, the co-culture suspension of *T. asperellum* 576 + *E. turcicum* 101 exhibited effective mycelial growth inhibition of *E. turcicum* 101 exceeding 50.00% from day 5 to 9. Similarly, the co-culture suspension of *T. atroviride* 393 + *E. turcicum* 101 showed effective mycelial growth inhibition exceeding 50.00% from days 6 to 9. These findings highlight the strong antifungal action of these co-culture suspensions, further emphasizing the potential of *T. asperellum* 576 and *T. atroviride* 393 as effective agents for inhibiting the growth of *E. turcicum* 101.

### 3.6. Activity of Cell Wall Degradation Enzymes

**Figure 6:**
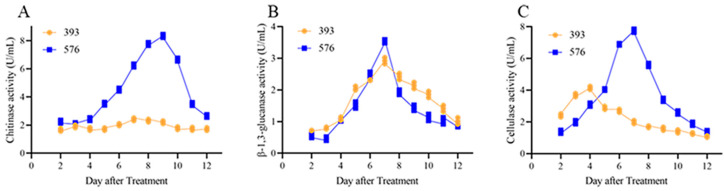
Cell wall degrading enzyme activities of the co-culture suspensions of *T. asperellum* 576 + *E. turcicum* 101 or *T. atroviride* 393 + *E. turcicum* 101. (**A**) chitinase; (B) β-1,3-glucanase; (**C**) cellulase. The length of the box represents the significance of the difference (*p* < 0.05).

The activity of cell wall degrading enzymes (chitinase, β-1,3-glucanase, and cellulase) in the co-culture suspensions of *T. asperellum* 576 + *E. turcicum* 101 or *T. atroviride* 393 + *E. turcicum* 101 was investigated. The chitinase activity in the co-culture suspension of *T. asperellum* 576 + *E. turcicum* 101 increased gradually until day 6 after treatment and reached its peak at day 9 with a value of 8.34 U/mL. On the other hand, the highest chitinase activity of 2.41 U/mL was observed on day 7 after treatment with the co-culture suspension of *T. atroviride* 393 + *E. turcicum* 101 (Figure 6A). The β-1,3-glucanase activity in the co-culture suspension of *T. asperellum* 576 + *E. turcicum* 101 also showed a gradual increase until day 3 after treatment and peaked at day 7 with a value of 3.42 U/mL. Similarly, the highest β-1,3-glucanase activity of 2.31 U/mL was detected on day 7 after treatment with the co-culture suspension of *T. atroviride* 393 + *E. turcicum* 101 (Figure 6B). As for cellulase activity, the co-culture suspension of *T. asperellum* 576 + *E. turcicum* 101 exhibited a gradual increase until day 6 after treatment and reached its peak at day 7 with a value of 7.75 U/mL. The highest cellulase activity of 4.14 U/mL was observed on day 4 after treatment with the co-culture suspension of *T. atroviride* 393 + *E. turcicum* 101 (Figure 6C). Comparatively, *T. asperellum* 576 showed stronger cell wall activities against *E. turcicum* 101 than *T. atroviride* 393. Therefore, *T. asperellum* 576 was selected for in vivo tests.

### 3.7. Stimulating Germination of Maize Seeds by T. asperellum 576

**Figure 7:**
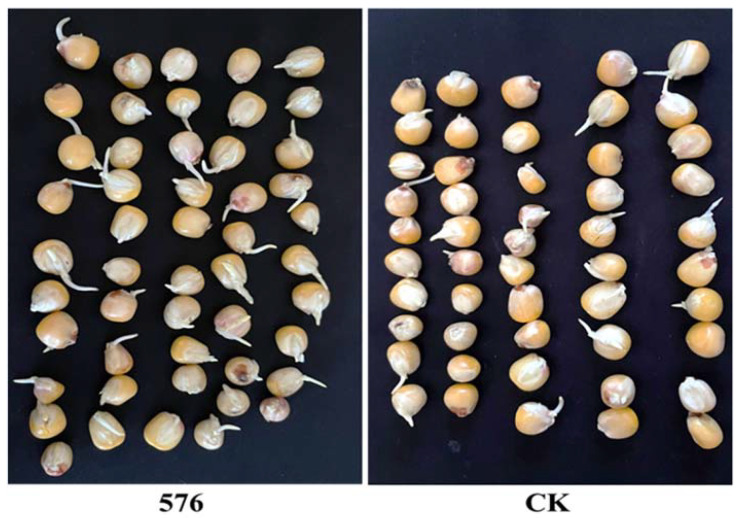
Effects of *Trichoderma asperellum* 576 treatment (**left**) on the germination of maize seeds in comparison with CK (**right**).

The impact of *T. asperellum* 576 conidial suspension on maize seed germination was evaluated compared to an untreated control group. *T. asperellum* 576 conidial suspension showed a significant promotion of seed germination compared to the inoculation control. As depicted in Figure 7, the seed germination rate of the control group was 50.90%. Among the *T. asperellum* 576 conidial suspension, there are forty-four maize seeds germinating, with rates reaching 91.00%. Based on these results, *T. asperellum* 576 was selected for further experiments.

### 3.8. Effect of T. asperellum 576 on E. turcicum 101 in Maize Seedlings

Under greenhouse conditions, the biocontrol effect of *T. asperellum* 576 against *E. turcicum* 101 on maize was evaluated. The performance of *T. asperellum* 576 in terms of plant growth promotion and suppression of *E. turcicum* 101 was assessed. These included four treatments: (T1) plants without *T. asperellum* 576 and *E. turcicum* 101 (healthy control), (T2) *T. asperellum* 576-treated plants, (T3) *E. turcicum* 101-inoculated plants (disease control), and (T4) *E. turcicum* 101 and *T. asperellum* 576-inoculated/treated plants.

**Figure 8:**
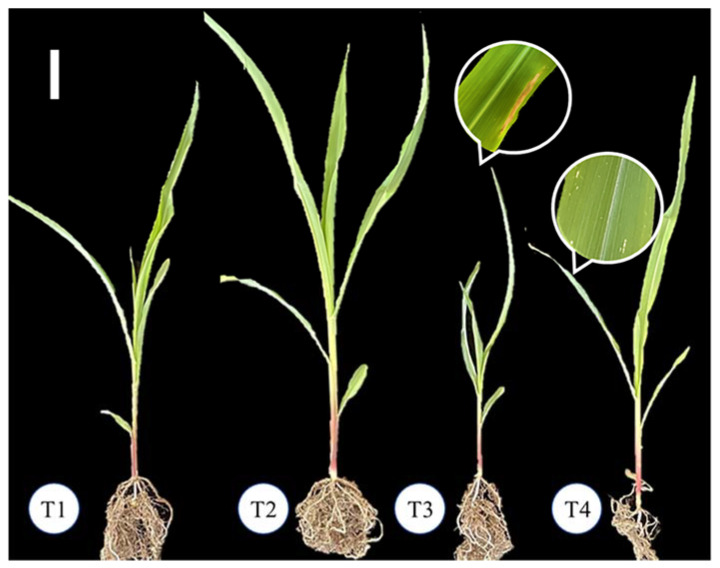
Effects of *Trichoderma asperellum* 576 on maize seedlings growth. T1: control plants (plants without *T. asperellum* 576 or *Exserohilum turcicum* 101; T2: *T. asperellum* 576-treated plants; T3: *E. turcicum* 101-inoculated plants; and T4: *E. turcicum* 101 and *T. asperellum* 576-inoculated/treated plants. Bar = 10 cm.

As shown in Table 5 and Figure 8, after 20 days, the maize seedlings treated with *T. asperellum* 576 exhibited significant improvements in shoot height, stem diameter, shoot fresh weight, root fresh weight, shoot dry weight, and root dry weight compared to the T1 control plants. The maize seedlings treated with *T. asperellum* 576 showed increases of 17.14%, 18.18%, 52.55%, 35.76%, 48.50%, and 47.27% in these parameters, respectively, indicating enhanced growth due to the presence of *T. asperellum* 576.

As shown in Table 5 and Figure 8, the maize seedlings inoculated with both *E. turcicum* 101 and *T. asperellum* 576 showed significant enhancements in shoot height, stem diameter, fresh and dry shoot weight, and fresh and dry root weight compared to the maize seedlings inoculated with *E. turcicum* 101. The promotion rates for these parameters in the maize seedlings inoculated with both *E. turcicum* 101 and *T. asperellum* 576 were 8.85%, 15.91%, 46.07%, 69.09%, 71.84%, and 94.52%, respectively. The blight of maize seedlings inoculated with both *E. turcicum* 101 and *T. asperellum* 576 was observed to be slighter than the maize seedlings inoculated with *E. turcicum* 101.

These results indicate that the application of *T. asperellum* 576 spore suspension effectively prevented the growth of *E. turcicum* 101 and promoted the growth of maize seedlings under greenhouse conditions.

## 4. Discussion

### 4.1. Evidence of Direct Mycoparasitism and Intraspecies Variability in Trichoderma-E. turcicum Interaction

In the dual culture test, a total of 12 strains of *Trichoderma* exhibited pronounced antagonistic activity. This phenomenon can potentially be attributed to the rapid growth rate of these *Trichoderma* strains in comparison to *E. turcicum* 101. *T. asperellum* 576 demonstrated the highest level of antifungal activity against *E. turcicum* 101 in the dual culture experiment. Moreover, we also observed significant variations between two *Trichoderma* strains belonging to the same species. This intraspecies variability aligns with findings reported by El Gamal et al. [53]. In order to comprehensively evaluate the impact of growth rate and mycoparasitism, we employed the evaluation methods described by Zhang et al. [47]. This method does not solely rely on assessing the inhibition percentage but instead considers both factors holistically, categorized as fast, moderate, or slow in comparison to pathogens. Consequently, these varying growth rates among *Trichoderma* strains may significantly influence the competition for nutrients and space, ultimately leading to the outcompeting of *E. turcicum* 101.

To gather additional evidence supporting direct mycoparasitism, a comprehensive analysis of microscopic images was conducted. A significant finding emerged wherein *T. asperellum* 576 hyphae were observed to encircle the hyphae of *E. turcicum* 101. These images were the mycelium of both *T. asperellum* 576 and *E. turcicum* 101. This discovery aligns with earlier research documenting physical contacts between hyphae, the abundant sporulation of *Trichoderma* on the hyphae of the pathogen, and the development of perforations when *Trichoderma* species interact with other pathogens [54,55]. Furthermore, the extensive literature confirms the species- and strain-dependent variability in the coiling behavior exhibited by different *Trichoderma* species. This observation provides additional support for the notion that the interaction between *Trichoderma* and its host is characterized by specificity [56,57].

### 4.2. Non-Volatile Metabolites of Trichoderma spp.: Potent Inhibitors of E. turcicum 101 and Antibiosis as a Key Biocontrol Mechanism

The antifungal activity of non-volatile metabolites derived from the tested *Trichoderma* spp. exhibited a significant growth inhibitory effect against *E. turcicum* 101. Previous studies have already established *T. asperellum* as an effective BCA against various plant-pathogenic fungi [58,59,60]. Consistent with these findings, our study demonstrated that *T. asperellum* 576 displayed strong inhibition of *E. turcicum* 101 growth (80.81% inhibition), and *T. atroviride* 393 also exhibited notable effectiveness against *E. turcicum* 101 (77.68% inhibition). Other *Trichoderma* species also exhibited varying degrees of radial growth inhibition against the tested pathogens, albeit to a lesser extent. Inhibitory action of these *Trichoderma* against the test pathogens primarily occurs through the production of non-volatile compounds, emphasizing the central role of antibiosis as the principal biocontrol mechanism [61]. Antibiosis refers to the antagonistic activity mediated by specific or non-specific metabolites of microbial origin, lytic enzymes, volatile compounds, and other toxic substances. It is noteworthy that *Trichoderma* spp. is known to synthesize various antibiotics, including trichodernin, trichodermol, and herzianolide, among others [62].

### 4.3. Unveiling the Pole of Trichoderma Volatile Substances in Inhibiting E. turcicum 101: A Potential Mechanism for Enhanced Biological Control

An additional potential mechanism that may contribute to the inhibition of *E. turcicum* 101 is the production of volatile substances. In our study, the results revealed significant suppressive effects, with *T. asperellum* 576-VOCs demonstrating the highest antifungal properties (65.86% inhibition) against *E. turcicum* 101. Conversely, *T. longibrachiatum* 3A displayed the lowest antifungal effect, with only 23.10%. These findings suggest that *Trichoderma* VOCs possess the potential to provide additional benefits in terms of biological control and promoting plant growth in sustainable agricultural practices. Notably, the inhibition rates of volatile metabolites do not necessarily correlate strongly with those of non-volatile metabolites, as the antagonistic effects involve various factors beyond the strain’s characteristics [63]. Previous research conducted by Amin et al. has shown that *Trichoderma*-derived VOCs can negatively affect the mycelial growth of various pathogenic fungi, including *Alternaria brassicicola*, *Rhizoctonia solani*, and *Fusarium oxysporum* [64]. Furthermore, several studies have documented the antifungal activity of *Trichoderma*-derived secondary metabolites. It is plausible that our *Trichoderma* strains also produce antifungal metabolites that diffuse through the agar, leading to a reduction in the mycelium growth of *E. turcicum* 101 [65,66]. To gain a deeper understanding, further investigations are required to isolate and purify the individual components present in these extracts and evaluate the spectrum of antifungal activity exhibited by these metabolites. Overall, it is likely that the observed inhibition of *E. turcicum* 101 is the result of a combination of different mechanisms at play involving both non-volatile and volatile compounds produced by *Trichoderma* strains.

### 4.4. Potent Antifungal Activity of T. asperellum 576 in Co-Culture with E. turcicum 101: Implications for Endophytic Environment Establishment

In this study, we conducted experiments using co-culture suspensions of *T. asperellum* 576 + *E. turcicum* 101 and *T. atroviride* 393 + *E. turcicum* 101 to assess their impact on the growth of *E. turcicum* 101. Notably, the *T. asperellum* 576 + *E. turcicum* 101 co-culture exhibited the most pronounced effect, demonstrating a significant broad-spectrum antifungal activity against *E. turcicum* 101 on the 7th day, resulting in a remarkable 64.45% inhibition. These findings strongly indicate the potent inhibitory effect of *T. asperellum* 576 on the growth of *E. turcicum* 101. Moreover, there is compelling evidence suggesting that the filtrates derived from these co-cultures may contain small plant-derived molecules that contribute to the establishment of an environment resembling a typical endophytic habitat [67]. Such an environment provides favorable conditions for the proliferation of microorganisms exhibiting endophytic behavior, including *Trichoderma* spp.

### 4.5. Role of Cell Wall-Degrading Enzymes in the Biocontrol Potential of T. asperellum 576 against E. turcicum 101

Continuing our investigation into the mechanisms involved in the biocontrol of *E. turcicum* 101, we conducted an evaluation of the chitinase, β-1,3-glucanase, and cellulase activities of *T. asperellum* 576. These enzymatic activities are known to play a critical role in the mycoparasitic activity exhibited by *Trichoderma* against pathogens. Our findings reveal that these cell wall-degrading enzymes do not act in isolation but require the concerted action of multiple chitinases to effectively break down fungal cell walls [68]. *Trichoderma* species have the ability to secrete hydrolytic enzymes that specifically target and degrade the cell walls of host fungi [24]. Extensive research has previously highlighted the remarkable capacity of the *Trichoderma* genus to produce a diverse range of hydrolytic enzymes and secondary metabolites, which contribute to the suppression of various pathogenic organisms [69]. Our study further demonstrated that the cell wall composition of *E. turcicum* 101 has a regulatory effect on the activities of *T. asperellum* 576 associated with mycoparasitism, including the production of β-1,3-glucanases, chitinases, and cellulase. These findings are consistent with the work of Aoki et al. [70], who reported the production and secretion of chitinase by *Trichoderma* sp. SANA20 in the culture media after three days of incubation. Moreover, our results align with previous studies that have shown significant chitinase and β-1,3-glucanase activities in the cell-free culture filtrate of *T. asperellum* [71,72,73]. Collectively, these findings suggest that *T. asperellum* 576 actively produces and secretes cell wall-degrading enzymes, which likely contribute to the suppression of fungal pathogens. The assessment of cell wall enzyme activities can serve as an indicator for determining antagonistic *Trichoderma* strains, with *T. asperellum* 576 exhibiting particularly high expression levels among the tested strains. This discovery holds significant importance in the identification of effective mycoparasites for biocontrol strategies.

### 4.6. Enhancing Maize Seed Germination with T. asperellum 576: Towards Optimal Spore Dose for Targeted Plant Growth Promotion

This study provides empirical support for the effect of applying *T. asperellum* 576 at a concentration of 10^7^ spores/mL on maize seeds, leading to a notable enhancement in seed germination compared to water-treated maize seeds (88% germination rate). These findings align with the observations made by Singh et al., who documented variations in the spore dose requirements of *T. asperellum* BHUT8 for promoting the initial growth of different vegetable crops, encompassing seed germination and radicle length [74]. In future investigations, our objective is to develop an innovative approach to determine the precise spore dose of *T. asperellum* 576 necessary to optimize plant growth on maize seeds. This research endeavor will yield valuable insights into refining the application of *T. asperellum* 576 for targeted plant growth promotion, specifically tailored to maize cultivation.

### 4.7. Exploring the Potential of T. asperellum 576 for Plant Growth Promotion and Disease Suppression

Previous research has extensively explored the direct effects of *Trichoderma*-based biopesticides or *Trichoderma* spore suspensions on various aspects of plant growth promotion, defense responses, and stress tolerance [75,76,77,78,79,80]. In greenhouse conditions, the application of *T. asperellum* 576 significantly enhanced the growth of maize seedlings (*p* < 0.05). These findings align with multiple studies indicating that the application of *Trichoderma* spp. leads to notable improvements in root length, shoot length, and dry weight compared to control plants [24,37,81]. One of the mechanisms attributed to the plant growth promotion capabilities of plant growth-promoting microorganisms’ strains is the production of growth-promoting hormones such as indole-3-acetic acid (IAA), cytokinins, and gibberellins, which stimulate plant growth [82]. In our study, we demonstrated that the treatment of maize seedlings with *T. asperellum* 576 resulted in increased fresh and dry weight, as well as lateral root length. These findings strongly suggest that *T. asperellum* 576 can effectively influence plant growth and dry matter accumulation. We speculate that the observed growth-promoting activity of plants might be mediated by hormones induced by *T. asperellum* 576. Future studies should investigate the reproducibility of the growth-promoting abilities of *T. asperellum* 576 under field conditions, following observations made in plants grown under greenhouse conditions with soil application. This line of research will provide valuable insights into the practical applicability of *T. asperellum* 576 as a plant growth promoter in real-world agricultural settings. Such knowledge is crucial for assessing its potential as a sustainable and effective tool for enhancing crop productivity.

In our investigation, the utilization of both *E. turcicum* 101 and *T. asperellum* 576 in treating maize seedlings observed a significant reduction in lesion area and an improved efficacy in controlling the impact of *E. turcicum* compared to seedlings treated solely with *E. turcicum* 101. These findings highlight the potential advantages of employing a combined treatment strategy, showcasing the synergistic effects of these two fungal strains in mitigating the detrimental consequences of *E. turcicum* 101 on maize plants. Interestingly, our study also revealed contrasting outcomes when maize seedlings were co-inoculated with *E. turcicum* 101 and *T. asperellum* 576. In this case, we observed a significant detrimental effect on the growth of the seedlings across various measured parameters, in contrast to the treatment with *E. turcicum* 101 alone. However, it is noteworthy that the maize seedlings subjected to the combined treatment of *E. turcicum* 101 and *T. asperellum* 576 displayed substantial improvements in shoot height, stem diameter, shoot fresh weight, root fresh weight, shoot dry weight, and root dry weight when compared to those treated solely with *E. turcicum* 101. These findings underscore the complexity of interactions among different microbial strains and their effects on plant health and growth. Further investigations are warranted to elucidate the underlying mechanisms driving the observed effects and to optimize the application strategies of these fungal strains for effective disease management in maize cultivation. In the current study, we observed remarkable growth inhibition of *E. turcicum* 101, the causal agent of northern corn leaf blight, in maize seedlings treated with *T. asperellum* 576. This effective control of the pathogen resulted in alleviating the symptoms associated with the disease. Previous studies have highlighted the potential of *T. asperellum* as a promising BCA against plant pathogens, and *T. asperellum* has been recognized for its capacity to enhance plant growth when introduced to plant roots, as demonstrated in a previous investigation [37,83,84,85,86]. The diverse biocontrol mechanisms demonstrated by *Trichoderma* spp. are pivotal in their efficacy for plant disease management and growth promotion across diverse contexts. Pathogen infections often induce significant morphological and physiological changes in plants, including the appearance of mosaic symptoms, which are closely linked to alterations in chlorophyll content and subsequent reductions in photosynthesis [87,88]. Consequently, our future investigations will assess changes in chlorophyll pigment content following *E. turcicum* 101 infection, thereby providing valuable insights into the physiological response of maize seedlings to pathogen-induced stress.

The current study provides compelling evidence supporting the effect of *T. asperellum* 576 isolates in suppressing the growth of *E. turcicum* 101 and inhibiting microsclerotia production while simultaneously promoting plant growth. Our findings contribute to a better understanding of the underlying mechanisms involved in the suppression of pathogenic activity by *E. turcicum* 101. These notable characteristics of the antagonistic strain can be effectively utilized in the development of future formulations and in preliminary experiments for the large-scale production of a bioinoculant containing *T. asperellum* 576 as the primary active component. We hypothesize that *Trichoderma* produces components that induce the synthesis of antimicrobial metabolites and cell wall degradation enzymes. This strategic approach can expedite and ensure the high efficiency of biocontrol when applied to maize plants. By synergistically combining the direct effects of pre-existing antimicrobial metabolites and cell wall degradation enzymes produced by *T. asperellum* 576 with the production of these components at the sites of microbial formulation application, the biocontrol activity can be significantly potentiated.

## 5. Conclusions

The study demonstrated the potential of *T. asperellum* 576 as a biocontrol agent against *E. turcicum* 101 and a growth promoter for maize. *T. asperellum* 576 showed strong antagonism and inhibitory effects on *E. turcicum* 101, both through non-volatile and volatile substances. It also exhibited enzymatic activities for degrading fungal cell walls. Additionally, *T. asperellum* 576 stimulated maize seed germination and enhanced plant growth. Overall, the findings suggest that *T. asperellum* 576 has promising potential as a biocontrol agent for disease management and as a bioenhancer for promoting the growth of maize.

## Figures and Tables

**Table 1:** Taxonomic information, strain/specimen number, and GenBank accession numbers for TEF1-α and RPB2 genes.

Taxon	Strain/Specimen	GenBank Accession Number	
		TEF1-α	RPB2
*Trichoderma atroviride*	393	**OR548045 ^1^**	**OR548096**
*T. afroharzianum*	XZ9-1	**OR548046**	**OR548111**
*T. breve*	578	**OR548049**	**-**
*T. brevicompactum*	592	**OR548083**	**OR548105**
*T. cerinum*	XZ1-3	**OR548077**	**OR548110**
*T. chromospermum*	91	**OR548054**	**-**
*T. chromospermum*	338	**OR548066**	**-**
*T. crassum*	110	KT149299	**-**
*T. guizhouense*	526	**OR548073**	**-**
*T. guizhouense*	TU2	**OR548076**	**OR548109**
*T. hamatum*	MSL-3	**OR548084**	**-**
*T. harzianum*	581	**OR548050**	**OR548103**
*T. koningiopsis*	421	**OR548081**	**OR548099**
*T. koningiopsis*	439	**OR548071**	**OR548101**
*T. linzhiense*	449	**OR548072**	**-**
*T. longibrachiatum*	539	**OR548074**	**OR548102**
*T. longibrachiatum*	3a	**OR548053**	**-**
*T. longifialidicum*	224	**OR548063**	**OR548091**
*T. longipile*	L-3	**OR548075**	**-**
*T. dorothopsis*	438	**OR548087**	**OR548100**
*T. pararogersonii*	219	**OR548062**	**-**
*T. paratroviride*	402	**OR548069**	**OR548097**
*T. paraviridescens*	295	**OR548079**	**OR548092**
*T. petersenii*	509	**OR548047**	**-**
*T. pleuroticola*	588	**OR548082**	**OR548104**
*T. polysporum*	1408	**OR548052**	**-**
*T. pyramidale*	285	**OR548064**	**-**
*T. rodmanii*	299	**OR548065**	**-**
*T. rodmanii*	376	**OR548080**	**OR548095**
*T. endophyticum*	99	KX689257	**OR548108**
*T. auriculariae*	417	**OR548070**	**OR548098**
*T. sinense*	204	**OR548060**	**-**
*T. solum*	375	**OR548068**	**OR548094**
*T. stipitatum*	218	**OR548061**	**-**
*T. strictipile*	370	**OR548078**	**-**
*T. strictipile*	115	**OR548058**	**OR548089**
*T. thailandicum*	1283	**OR548086**	**OR548090**
*T. thelephoricola*	342	**OR548067**	**OR548093**
*T. tomentosum*	153	**OR548059**	**-**
*T. vinosum*	XZ5-2	**OR548085**	**-**
*T. longibrachiatum*	593	**OR548051**	**OR548106**
*T. zonatum*	220	MF374809	MF374806
*T. italicum*	64	OR548054	**OR548107**
*T. asperellum*	576	**OR548047**	**OR548088**
*T. asperellum*	HZA10	MK850832	MH647800
*T. atroviride*	CBS 119499	FJ860611	FJ860518
*T. breve*	HMAS248845	KY688046	KY687984
*T. compactum*	CBS 121218	KF134798	KP115276
*T. evansil*	Dis 282d	EU856319	FJ150784
*T. helicolixii*	CBS 133499	KJ665517	KJ665278
*T. italicum*	CBS 132567	KJ665525	KJ665282
*T. harzianum*	CBS 226.95	AF534621	AF545549
*T. guizhouense*	S628	KJ665511	KJ665273
*T. hamatum*	Th23	OL439486	OL412667
*T. longibrachiatum*	CBS 816.68	AY865640	DQ087242
*T. polysporum*	CPK 3131	FJ860661	JQ685878
*T. rodmanii*	CBS 121553	FJ860687	FJ860580
*T. koningiopsis*	GJS 93-20	DQ284966	EU241506
*T. paratroviride*	CBS136489	KJ665627	KJ665321
*T. brevicompactum*	CBS 109720	OP203936	OP203935
*T. pseudolacteum*	TUFC 61490	JX238493	JX238478

^1^ Newly generated sequences in this study are indicated in bold, and sequences that are unavailable in GenBank are indicated by “-”.

**Table 2:** Inhibition effect of non-volatile substances of *Trichoderma* strains against *Exserohilum turcicum* 101.

Strains	Colony Diameter (mm)	Inhibition Rate (%)
576	11.18	80.81 ± 2.01 a ^1^
393	13.29	77.68 ± 3.87 ab
421	15.84	73.41 ± 2.70 ab
110	18.35	69.20 ± 1.23 ab
3A	20.35	65.84 ± 5.60 bc
XZ9-1	27.64	53.60 ± 4.36 cd
285	27.95	53.07 ± 2.94 d
539	27.95	53.07 ± 2.94 d
417	34.42	42.22 ± 2.02 de
XZ1-3	37.25	37.46 ± 2.44 ef
342	40.21	32.49 ± 3.01 ef
TU2	40.64	31.77 ± 5.10 ef
64	43.92	26.27 ± 3.30 fg
402	44.82	24.75 ± 6.14 g
CK	59.56	-

^1^ Means ± SD followed by the same letters do not differ from each other according to the Fisher’s test (*p* < 0.05).

**Table 3:** Inhibition effect of volatile substances of *Trichoderma* strains against *Exserohilum turcicum* 101.

Strain	Colony Diameter (mm)	Inhibition Rate (%)
576	14.72	65.86 ± 0.27 a ^1^
393	18.31	57.54 ± 0.71 ab
421	19.9	53.85 ± 0.34 b
110	29.12	32.47 ± 0.75 c
3A	33.16	23.10 ± 0.83 d
CK	43.12	-

^1^ Means ± SD followed by the same letters do not differ from each other according to the Fisher’s test (*p* < 0.05).

**Table 4:** Inhibitory effect of the co-culture suspension on *Exserohilum turcicum* 101.

Day	*T. asperellum* 576 + *E. turcicum* 101		*T. atroviride* 393 + *E. turcicum* 101	
	Colony Diameter (mm)	Inhibition Rate (%)	Colony Diameter (mm)	Inhibition Rate (%)
2	48.79	12.48 ± 2.69 gh ^1^	41.53	25.50 ± 3.53 h
3	40.18	27.93 ± 4.32 e	36.86	34.12 ± 2.13 fg
4	31.07	44.26 ± 3.42 cd	31.90	43.02 ± 1.95 e
5	28.19	50.43 ± 3.63 bc	30.20	46.22 ± 1.41 de
6	25.85	53.63 ± 0.64 b	20.94	62.50 ± 4.95 a
7	19.82	64.45 ± 2.52 a	27.69	50.50 ± 0.71 cd
8	34.04	54.94 ± 3.15 b	24.08	57.00 ± 1.39 b
9	44.09	50.91 ± 4.23 bc	25.75	54.03 ± 1.88 bc
10	46.41	39.74 ± 0.84 de	28.40	49.25 ± 2.37 cd
11	47.20	15.32 ± 0.86 fg	35.29	36.50 ± 2.12 f
12	51.84	10.71 ± 3.78 h	38.27	31.14 ± 2.83 g
CK	55.74	-	-	-

^1^ Means ± SD followed by the same letters do not differ from each other according to the Fisher’s test (*p* < 0.05).

**Table 5:** Effect of *Trichoderma asperellum* 576 on maize seedlings growth. T1: control seedlings without *T. asperellum* 576 or *Exserohilum turcicum* 101; T2: maize seedlings treated with *T. asperellum* 576; T3: maize seedlings inoculated with *E. turcicum* 101; and T4: maize seedlings inoculated/treated with *E. turcicum* 101 and *T. asperellum* 576.

Treatment	Shoot Height (cm)	Stem Diameter (cm)	Fresh Shoot (g)	Fresh Root (g)	Dry Shoot (g)	Dry Root (g)
T1	70.00 ± 2.00 b ^1^	0.55 ± 0.03 b	9.42 ± 1.18 b	3.44 ± 0.40 b	2.00 ± 0.15 b	1.65 ± 0.18 b
T2	82.00 ± 1.73 a	0.65 ± 0.00 a	14.37 ± 1.86 a	4.67 ± 0.55 a	2.97 ± 0.26 a	2.43 ± 0.06 a
T3	60.33 ± 1.53 c	0.44 ± 0.05 c	5.17 ± 1.26 c	1.65 ± 0.67 c	1.03 ± 0.49 c	0.73 ± 0.40 c
T4	65.67 ± 3.21 b	0.51± 0.20 b	7.50 ± 0.50 c	2.79 ± 0.24 b	1.77 ± 0.17 b	1.42 ± 0.19 b

^1^ Means ± SD followed by the same letters do not differ from each other according to the Fisher’s test (*p* < 0.05).

## Data Availability

Not applicable.

## Supplementary Materials

The following supporting information can be downloaded at: https://www.mdpi.com/article/10.3390/jof9090936/s1, Figure S1: Forty-four *Trichoderma* strains used in this study cultures at 25 °C after 7 days; Figure S2: *Trichoderma* strains with moderate antagonistic (left) and *E. turcicum* 101 (right) strains in dual culture. 1–3: 110; 4–6: 224; 7–9: 295; 10–12: 375; 13–15: 99; 16–18: 421; 19–21: 539; 22–24: 581; 25–27: 593; 28–30: 526; 31–33: 578; 34–36: XZ9-1; Figure S3: *Trichoderma* with weakly antagonistic (left) and *E. turcicum* 101 (right) strains in dual culture. 1–3: 153; 4–6: 218; 7–9: 219; 10–12: 220; 13–15: 370; 16–18: 439; 19–21: 588; 22–24: 1408; 25–27: L-3; 28–30: 592; Figure S4: *Trichoderma* without antagonistic (left) and *E. turcicum* 101 (right) strains in dual culture. 1–3: 115; 4–6: 299; 7–9: 338; 10–12: 376; 13–15: 438; 16–18: 449; 19–21: 509; 22–24: 1283; 25–27: XZ5-2, 28-30: 91.
