# Supplementary material for: Biocontrol Potential of Trichoderma asperellum Strain 576 against Exserohilum turcicum in Zea mays
Source: J Fungi (Basel). 2023 Sep 16;9(9):936. doi: 10.3390/jof9090936 (PMC10532967; doi:10.3390/jof9090936)
Supplement: Supplementary file 1 [file jof-09-00936-s001.zip › jof-2504630-supplementary.pdf]

Supplementary Materials

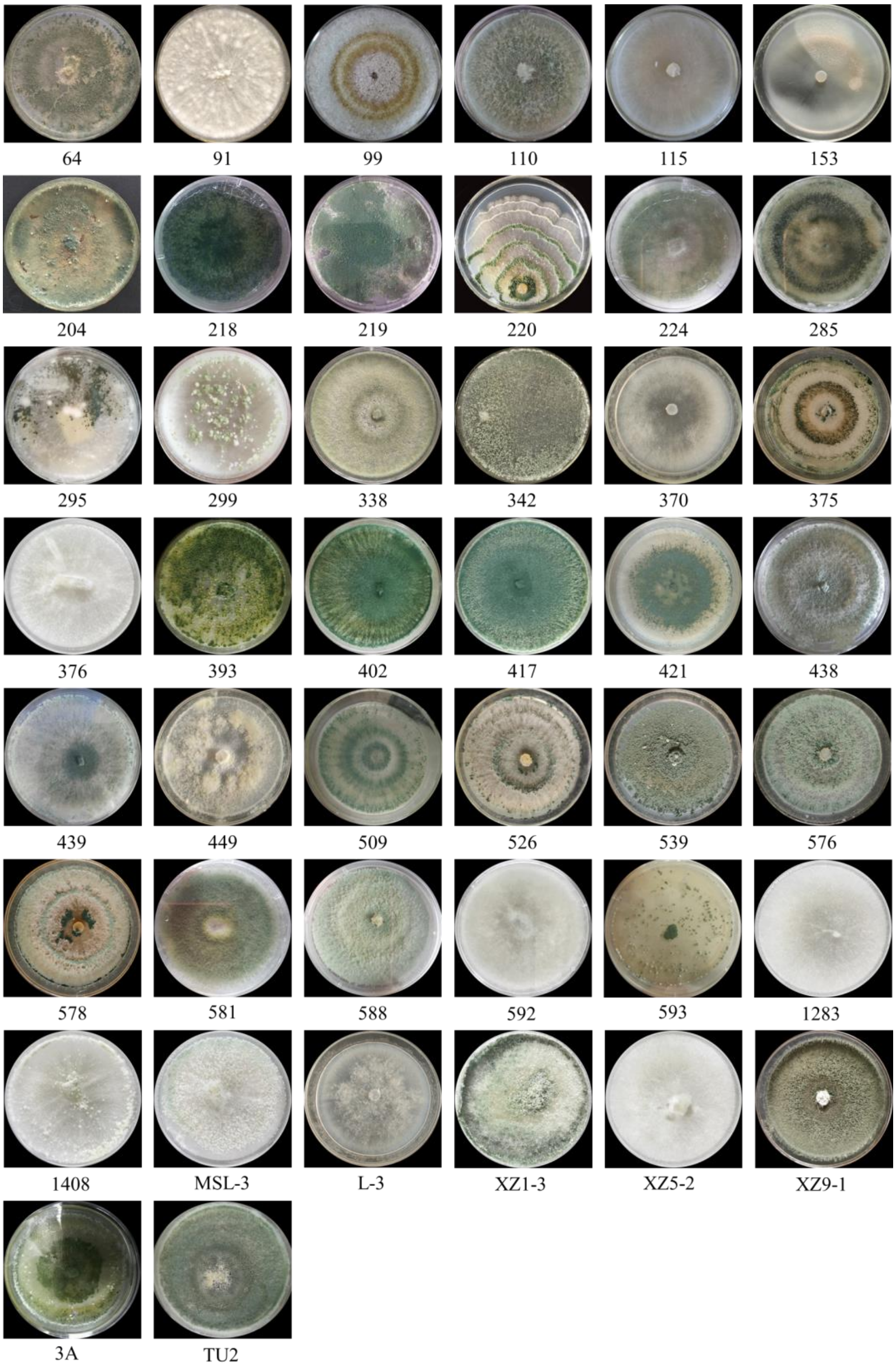

Figure S1. Forty-four *Trichoderma* strains used in this study cultures at 25 °C after 7 days.

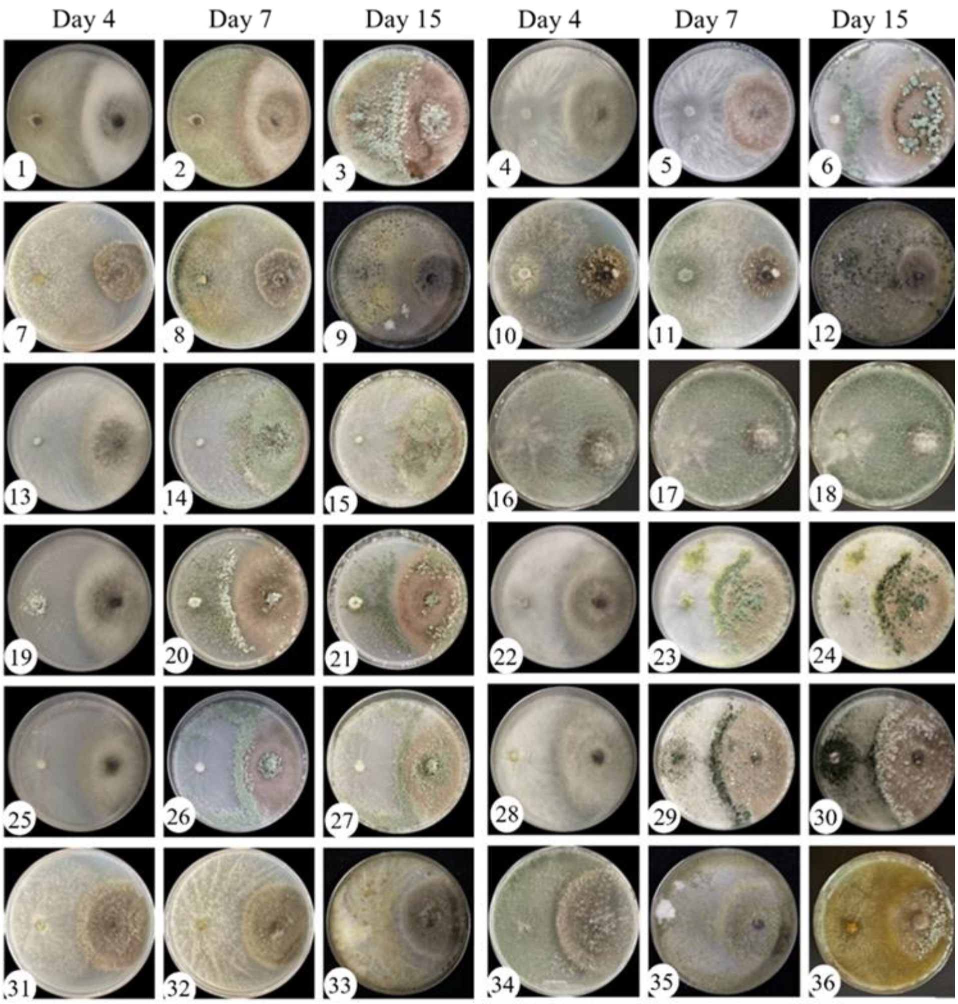

Figure S2. *Trichoderma* strains with moderate antagonistic (left) and *E. turcicum* 101 (right) strains in dual culture. 1–3: 110; 4–6: 224; 7–9: 295; 10–12: 375; 13–15: 99; 16–18: 421; 19–21: 539; 22–24: 581; 25–27: 593; 28–30: 526; 31–33: 578; 34–36: XZ9-1.

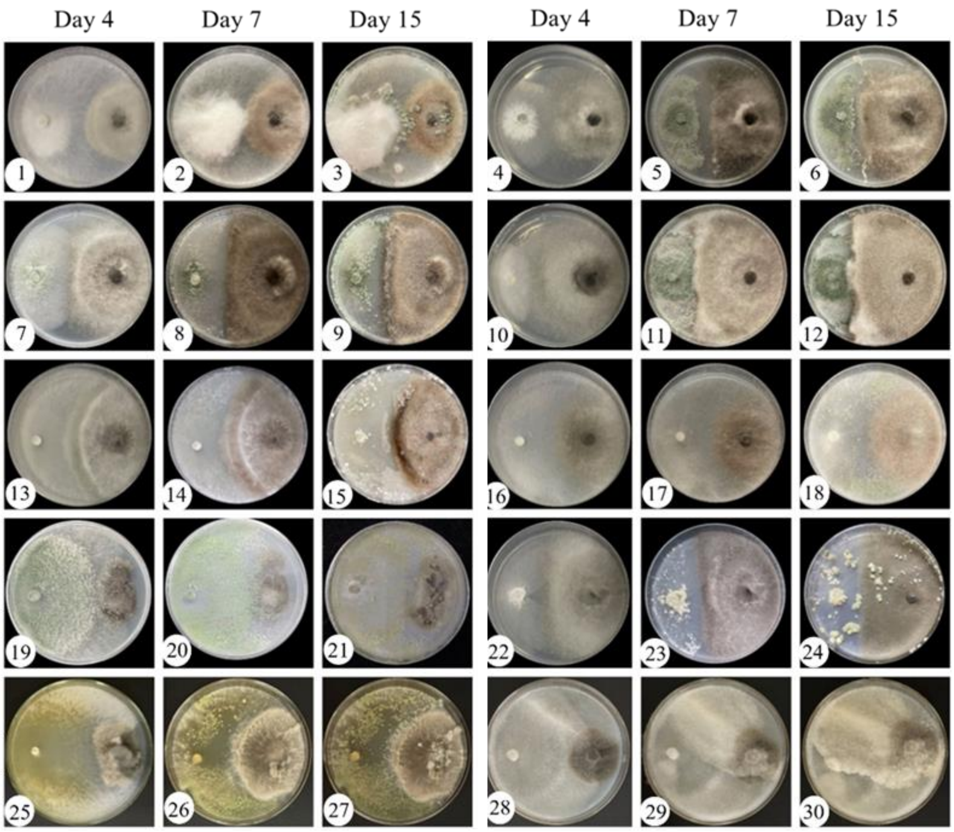

Figure S3. *Trichoderma* with weakly antagonistic (left) and *E. turcicum* 101 (right) strains in dual culture. 1–3: 153; 4–6: 218; 7–9: 219; 10–12: 220; 13–15: 370; 16–18: 439; 19–21: 588; 22–24: 1408; 25–27: L-3; 28–30: 592.

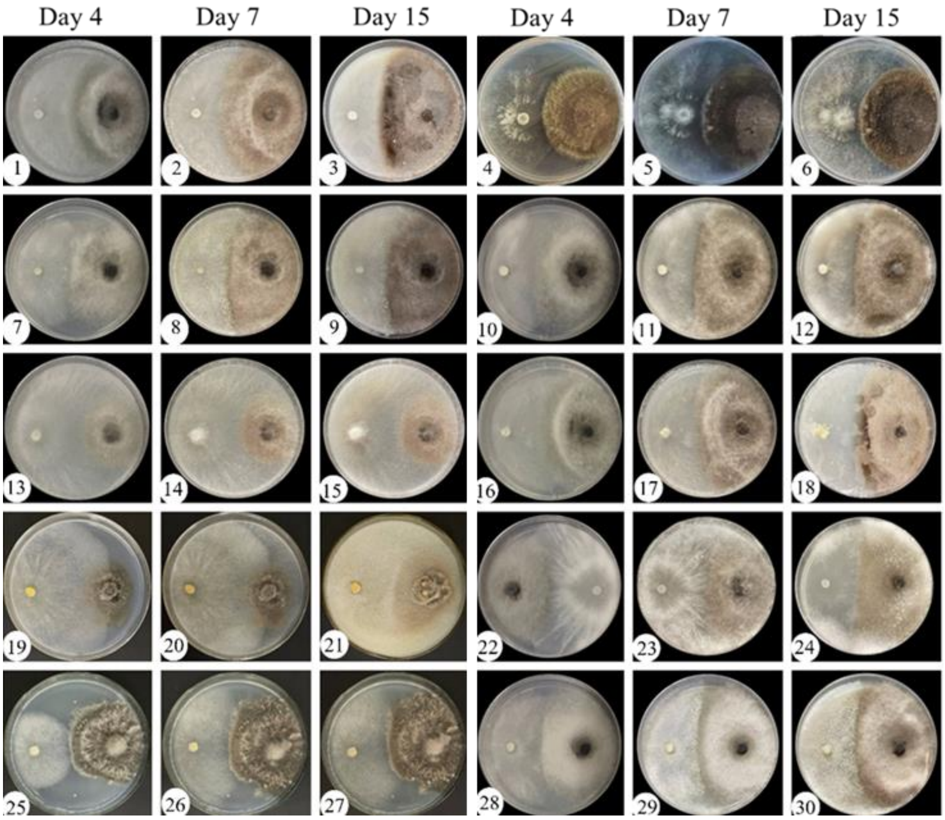

Figure S4. *Trichoderma* without antagonistic (left) and *E. turcicum* 101 (right) strains in dual culture. 1–3: 115; 4–6: 299; 7–9: 338; 10–12: 376; 13–15: 438; 16–18: 449; 19–21: 509; 22–24: 1283; 25–27: XZ5-2, 28-30: 91.
